# Fabrication of a BC/1T-2H MoS_2_@TP/PPy Membrane for Photo-Fenton Reactions and Solar-Driven Water Evaporation

**DOI:** 10.3390/molecules31162878

**Published:** 2026-08-18

**Authors:** Bochen Jiang, Xiaojing Sui, Ziran Niu, Yanhua Lei, Jie Wang, Liu Hui, Bing Zhang, Jiangdong Cao, Yu Gu, Zhongzhou Cui

**Affiliations:** 1 School of Intelligent Manufacturing and Information, Jiangsu Shipping College, Nantong 226010, China; bochenjiang@hotmail.com (B.J.); caojd@jssc.edu.cn (J.C.); gy15206278955@hotmail.com (Y.G.); c15152286019@hotmail.com (Z.C.); 2 College of Foreign Languages, Shanghai Maritime University, Shanghai 201306, China; xisui@shmtu.edu.cn; 3 College of Ocean Science and Engineering, Shanghai Maritime University, Shanghai 201306, China; 202430410112@stu.shmtu.edu.cn (Z.N.); liang.zai.la@outlook.com (L.H.); 4 School of Materials Science and Engineering, Central South University, Changsha 410083, China; m18805485953@163.com; 5 Zhejiang Key Laboratory of Intelligent Manufacturing for Functional Chemicals, ZJU-Hangzhou Global Scientific and Technological Innovation Center, Zhejiang University, Hangzhou 311215, China

**Keywords:** bacterial cellulose, 1T-2H MoS_2_, photo-Fenton, photothermal water evaporation

## Abstract

Solar-driven advanced oxidation processes and interfacial water evaporation represent promising strategies to simultaneously address water pollution and freshwater scarcity. However, conventional photo-Fenton catalysts are predominantly powder-based and suffer from difficult catalyst recovery, inefficient H_2_O_2_ utilization, and limited practical applicability. Herein, a multifunctional bacterial-cellulose-based composite membrane (BC-MTP) was designed by immobilizing 1T-2H MoS_2_ and a tea-polyphenol/polypyrrole (TP/PPy) copolymer through a blending–copolymerization strategy. The three-dimensional BC network anchors the catalytic components and supports their repeated use, while the BC-MTP membrane achieves 88.2% tetracycline removal using 20 μL H_2_O_2_, four times lower than the dosage used for the corresponding powder catalyst. Because dissolved Fe was not quantified by ICP-OES or ICP-MS, the present results are interpreted as evidence of improved H_2_O_2_ utilization and component immobilization rather than quantitative proof of suppressed iron leaching. The incorporation of 1T-2H MoS_2_ and TP/PPy also provides strong broadband light absorption and photothermal conversion, yielding a solar-driven evaporation rate of 1.34 kg m^−2^ h^−1^ and an energy efficiency of 80.1% under the tested laboratory conditions. This work demonstrates a membrane platform integrating photo-Fenton degradation and solar-driven water evaporation for water-purification applications.

## 1. Introduction

To better address challenges such as water pollution, water scarcity, and the energy crisis, it is particularly crucial to develop sustainable and energy-efficient green water treatment technologies to ensure access to clean water resources [1,2]. In current research, most catalysts used for photocatalytic Fenton degradation exist in powder form [3,4,5]. However, the recovery of powder catalysts after the catalytic degradation process has been a major challenge hindering their commercialization in industrial applications.

The photocatalytic Fenton degradation process primarily relies on iron as a catalyst to enhance the oxidative capability of H_2_O_2_ [6,7,8]. In previous studies [9], we successfully addressed the issue of external iron sources by introducing tea polyphenols as an iron source. The prepared composite catalyst demonstrated excellent photocatalytic Fenton degradation performance. Nevertheless, the recovery of the composite catalyst remains a significant limiting factor in its potential for industrial application.

In photocatalytic Fenton degradation technology, researchers have proposed a strategy of using ferromagnetic materials as catalysts, aiming to address the issue of difficult recovery associated with traditional powder catalysts [10,11,12]. However, this approach may pose the issue of iron leaching. If the leaching rate of iron is too rapid, it can not only lead to the formation of iron sludge [13,14,15] but also potentially cause excessive consumption of hydrogen peroxide (H_2_O_2_) [16,17,18].

Immobilizing a catalyst on a suitable substrate can facilitate catalyst recovery and reduce the rapid physical loss of powdered active components during repeated operation [19,20,21]. Nevertheless, immobilization alone does not quantitatively demonstrate suppression of dissolved-metal leaching; such a conclusion requires direct analysis of the post-reaction solution. Bacterial cellulose (BC), a biopolymer with high porosity, hydrophilicity, and water-retention capacity, is a promising flexible support for anchoring functional materials [22,23]. BC-based nanocomposites can be fabricated through biosynthetic or post-synthetic modification routes [24] and have attracted attention for adsorption and photocatalytic water treatment [25,26,27].

To utilize solar energy more comprehensively, multifunctional evaporators that combine photothermal conversion with pollutant removal have emerged as an important direction. Recent studies emphasize that high evaporation rates alone are insufficient for practical deployment; broadband absorption, structural and chemical stability, salt resistance, water transport, and performance in realistic water matrices must also be considered [28,29]. In particular, coupling photo-Fenton catalysis with interfacial evaporation offers a route to limit the enrichment or carryover of organic pollutants during solar steam generation [30,31].

In this work, bacterial cellulose was used as a flexible porous substrate to immobilize 1T-2H MoS_2_ and a tea-polyphenol/polypyrrole (TP/PPy) copolymer through blending and in situ copolymerization. The BC framework provides physical confinement of the functional components and promotes water transport. The 1T-2H MoS_2_ and TP/PPy components contribute to light harvesting, photothermal conversion, and H_2_O_2_ activation. Because equivalent electronic-structure calculations for the individual components were not available, enhanced charge separation is discussed as a possible interfacial effect rather than a theoretically proven consequence of bandgap narrowing. Catalytic activity, H_2_O_2_ dosage, temperature-associated photo-Fenton enhancement, cyclic performance, and laboratory water-evaporation performance were evaluated. The results establish a proof-of-concept platform for integrated water purification and freshwater generation, while long-term seawater desalination performance remains to be verified.

## 2. Results and Discussion

### 2.1. Morphology and Structural Characterizations

**Figure 1:**
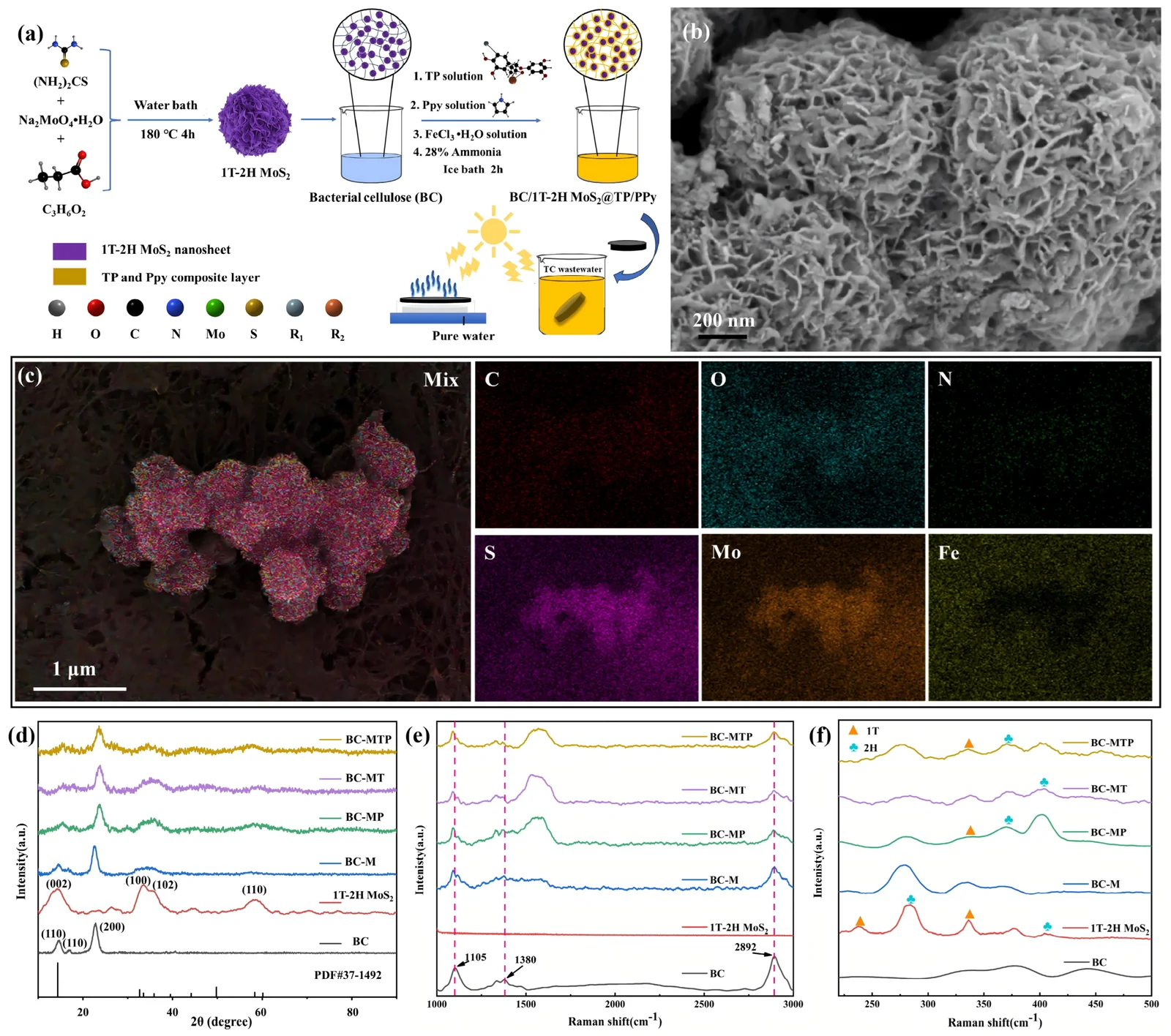
Morphology and structural analysis. (**a**) Schematic illustration for the preparation of BC-MTP composite membrane material; (**b**) SEM Images and (**c**) EDS Spectrum of BC-MTP; XRD Patterns (**d**) and Raman Spectra (**e**,**f**) of BC, 1T-2H MoS_2_, BC-M, BC-MP, BC-MT, and BC-MTP, respectively.

The typical flower-like structure of 1T-2H MoS_2_ can be well preserved even after the loading of the copolymer, as shown in Figure 1b and Figure S1. Furthermore, the presence of the network-like fibers and nanoflower structure indicates that, through the blending method, the nanoflower-like 1T-2H MoS_2_ was successfully integrated with BC, forming an interwoven structure. However, a potential issue with this blending method is the tendency for 1T-2H MoS_2_ powder to agglomerate, which is also confirmed by the energy-dispersive X-ray spectroscopy (EDS) results (Figure 1c) for Mo and S elements. Additionally, the presence of Fe and N elements in the EDS spectra further confirms the successful incorporation of tea polyphenols and PPy into the composite material. Then, XRD characterization was employed to systematically analyze the crystal structure of the composite membrane materials. Figure 1d presents the XRD results of six different materials: BC, 1T-2H MoS_2_, BC-M, BC-MP, BC-MT, and BC-MTP. The characteristic diffraction peaks at 14.8°, 16.75°, and 22.82° correspond to the (110), (110), and (200) crystal planes of bacterial cellulose, respectively. The presence of these peaks confirms the cellulose I crystalline structure [32]. Further analysis of the XRD patterns of BC-M, BC-MP, BC-MT, and BC-MTP composite membrane materials reveals that all these materials retain the three characteristic diffraction peaks of BC mentioned above. This indicates that the loading of catalyst materials did not affect the crystalline state of BC. Notably, in the BC-MP, BC-MT, and BC-MTP composite membranes, a slight rightward shift of the three characteristic diffraction peaks of BC is observed. This phenomenon may be associated with the polymerization reaction occurring under alkaline conditions. Additionally, the characteristic diffraction peaks at 14.4°, 33.2°, 35.6°, and 58.2° correspond to the (002), (100), (102), and (110) crystal planes of 1T-2H MoS_2_, respectively. These characteristic peaks are also observed in the BC-MP, BC-MT, and BC-MTP composite membranes. Based on the above analysis, it can be concluded that 1T-2H MoS_2_ was successfully loaded onto the BC substrate via the blending method, and the copolymerization reaction did not significantly affect the crystalline structures of either 1T-2H MoS_2_ or BC.

Raman spectra were also applied to further reveal the structure of composite materials, as shown in Figure 1e; the characteristic peak at 1105 cm^−1^ corresponds to the asymmetric stretching vibration of the C-O-C glycosidic bond in BC. The peak at 1380 cm^−1^ is attributed to the bending vibration of C-H in BC, while the peak at 2892 cm^−1^ is associated with the stretching vibration of C-H/CH_2_ groups in BC [33]. Additionally, in the 1000–3000 cm^−1^ range, no characteristic peaks from 1T-2H MoS_2_ are observed. Furthermore, the three typical characteristic peaks of BC are present in the Raman spectra of BC-M, BC-MP, BC-MT, and BC-MTP, indicating that the loading of the catalytic materials did not alter the basic structure of BC, which is consistent with the XRD data. After loading the catalytic components, the BC-related Raman bands exhibit lower intensities in the composite membranes. This attenuation should not be regarded as direct evidence of alkaline degradation of the BC framework, because Raman intensity may also be affected by surface coverage of the BC fibers by the strongly absorbing MoS_2_/TP/PPy phase, optical attenuation, sampling-position differences, local compositional heterogeneity, and possible changes in crystallinity. Therefore, the Raman results are used here only to confirm the retention of the principal vibrational features of BC. Figure 1f shows the crystal phase analysis of 1T-2H MoS_2_. In the BC-M, BC-MP, BC-MT, and BC-MTP samples, characteristic peaks corresponding to the 1T and 2H phases of MoS_2_ are observed. The peaks at 377 cm^−1^ and 402 cm^−1^ correspond to the 2H-MoS_2_ phase, while the peaks at 238 cm^−1^, 284 cm^−1^, and 336 cm^−1^ correspond to the 1T-MoS_2_ phase. This indicates the successful introduction of the metal 1T phase into MoS_2_, and that the presence of the BC substrate and the occurrence of the copolymerization reaction did not alter the phase composition of MoS_2_.

**Figure 2:**
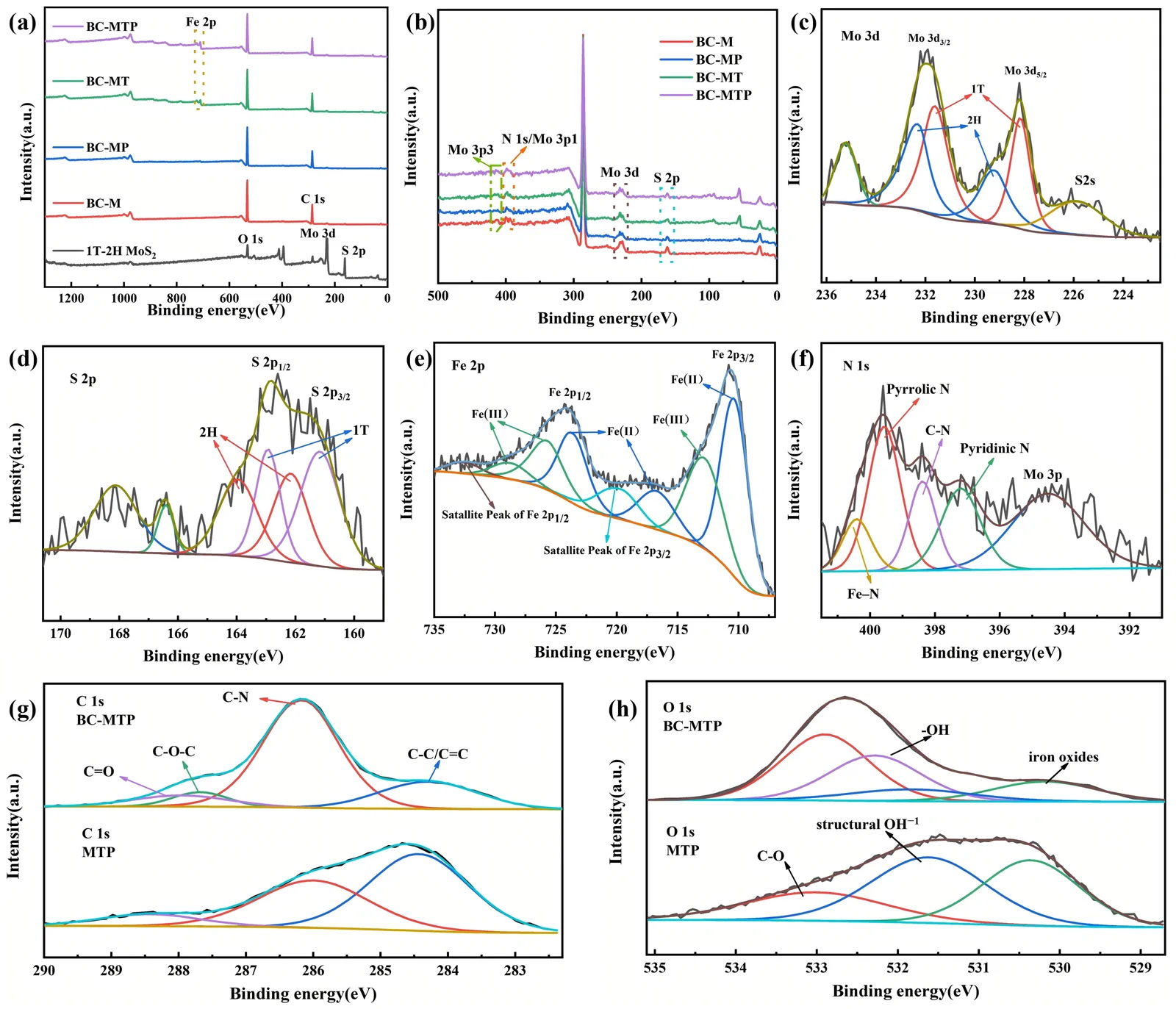
XPS analysis. (**a**,**b**) XPS full spectra of 1T-2H MoS_2_, BC-M, BC-MP, BC-MT, BC-MTP, and high-resolution XPS spectra of BC-MTP: (**c**) Mo 3d, (**d**) S 2p, (**e**) Fe 2p, (**f**) N 1s. XPS high-resolution spectra of BC-MTP and MTP: (**g**) C 1s and (**h**) O 1s.

To further analyze the elemental composition and chemical states of the composite membrane materials, XPS characterization (Figure 2, Tables S1 and S2) was performed. As shown in Figure 2a,b, the presence of Fe, Mo, and S elements in the BC-MT and BC-MTP composite membrane materials indicates the successful incorporation of tea polyphenols and 1T-2H MoS_2_. In the Mo 3d spectrum, four characteristic peaks are observed (Figure 2c). After deconvolution, the peaks at 228.2 eV and 231.6 eV correspond to the 1T phase of MoS_2_, while the peaks at 229.3 eV and 232.3 eV are attributed to the 2H phase of MoS_2_ [34,35]. Additionally, the peak at 225.8 eV corresponds to S 2s in 1T-2H MoS_2_, and the peak at 235.2 eV is associated with Mo-O, indicating the presence of MoO_3_ within the 1T-2H MoS_2_ structure. However, the corresponding XRD results do not show characteristic diffraction peaks of MoO_3_. This suggests that the presence of MoO_3_ in 1T-2H MoS_2_ might be due to oxidation caused by prolonged storage in air [36]. Figure 2d presents the high-resolution S 2p spectrum of the BC-MTP composite membrane. Two distinct peaks observed at 161.2 eV and 162.9 eV are attributed to the 1T phase of MoS_2_, while another two peaks at 162.2 eV and 164.1 eV correspond to the 2H phase of MoS_2_. Additionally, the peak located at 166.3 eV likely originates from the characteristic signal of SO_3_^−^. Notably, the peak at 168.1 eV is attributed to the partial oxidation of sulfur on the surface of 1T-2H MoS_2_ [34]. The presence of these characteristic peaks provides direct evidence for the different phases of MoS_2_ and its surface chemical states within the BC-MTP material.

In the high-resolution Fe 2p spectrum (Figure 2e), the peaks at 710.5 eV, 716.79 eV, and 723.8 eV correspond to the characteristic signals of Fe^2+^, while the peaks at 713 eV, 725.9 eV, and 728.9 eV are attributed to the characteristic signals of Fe^3+^ [37,38]. Additionally, the peaks at 720 eV and 732.6 eV correspond to the satellite peaks of Fe 2p_3/2_ and Fe 2p_1/2_, respectively. These findings indicate the presence of both Fe^2+^ and Fe^3+^ oxidation states in the BC-MTP membrane material. Figure 2f shows the high-resolution N 1s spectrum. Four distinct peaks are observed at 397.21 eV, 398.4 eV, 399.57 eV, and 400.4 eV, corresponding to pyridinic-N, C-N bonds, pyrrolic-N, and iron-nitrogen bonds (Fe-N type) [34,39], respectively. The presence of pyridinic-N and pyrrolic-N confirms the successful polymerization of PPy. The presence of Fe-N bonds further confirms the successful incorporation of TP and the formation of TP-iron coupled structures. Additionally, the peak at 394.54 eV is attributed to Mo 3p.

As shown in Figure 2g, the C 1s spectrum of BC-MTP exhibits four characteristic peaks. The peaks at 284.32 eV, 286.18 eV, and 288.09 eV correspond to C–C/C=C bonds, C–N bonds, and C=O bonds, respectively, as observed in MTP. The presence of the C=O bond further confirms the successful loading of TP. However, a partial shift in binding energy is observed in BC-MTP, which can be attributed to the non-conductive nature of the BC-MTP membrane. During XPS analysis, this shift is likely caused by charge accumulation or dissipation. Additionally, a new characteristic peak at 287.68 eV is observed, which can be attributed to the C–O–C bond in BC. This is consistent with some of the results from the Raman tests. Figure 2h shows the O 1s high-resolution spectra of BC-MTP and MTP. The O 1s analysis of MTP indicates three distinct peaks at 530.35 eV, 531.63 eV, and 533.06 eV, which correspond to iron oxides, structural OH^−1^ groups, and C–O bonds, respectively [40]. In the O 1s spectrum of BC-MTP, four characteristic peaks appear. The binding energies corresponding to iron oxides, structural OH^−1^ groups, and C–O bonds shift to 529.85 eV, 531.57 eV, and 532.75 eV, which may be due to the same reasons that caused the binding energy shift in the C 1s spectrum. Additionally, the extra peak at 532.12 eV could correspond to hydroxyl groups (–OH) in BC. These results indicate the successful incorporation of TP and the presence of BC in the composite membrane material.

### 2.2. Photothermal Fenton Performance Analysis

To evaluate the catalytic performance of the BC-MTP composite membrane material in the photo-Fenton process, tetracycline (TC) was used as a model pollutant for comprehensive performance assessment. The degradation rate of different materials can be compared using the first-order kinetics fitting formula [41,42]:

(1) $$\ln\left(\frac{C_{0}}{C_{t}}\right)=kt$$

where *C*_0_ and *C_t_* are the initial concentration of the pollutant and the concentration after the preselected exposure time, respectively; *k* (min^−1^) is the degradation rate constant; and *t* is the sunlight exposure time (minutes).

**Figure 3:**
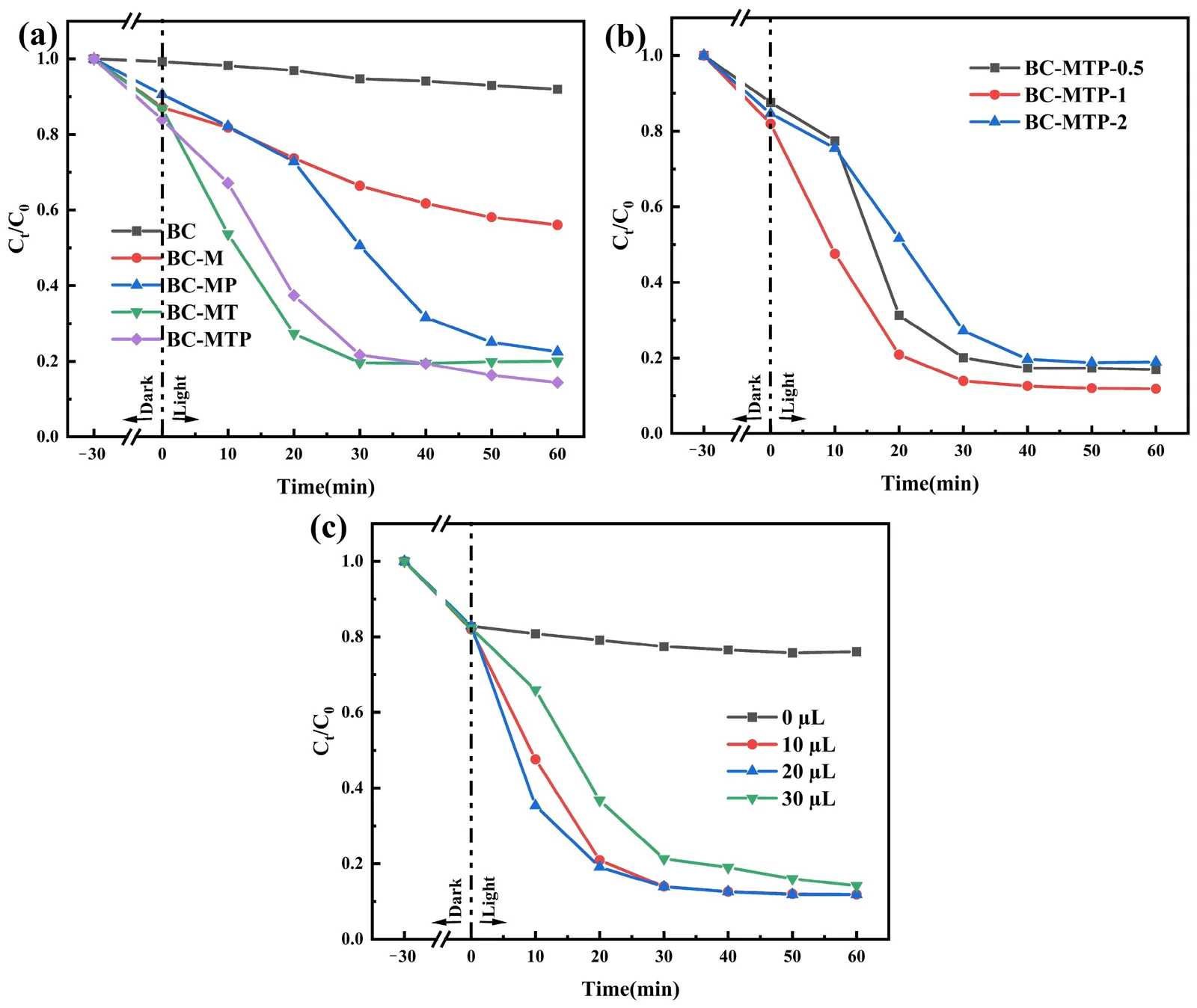
(**a**) Photocatalytic-Fenton degradation of TC by different membrane materials; (**b**) Photocatalytic-Fenton degradation of TC by BC-MTP membrane under different loading amounts; (**c**) Photocatalytic-Fenton degradation of TC by BC-MTP membrane under different H_2_O_2_ addition amounts.

The catalytic performance of different membrane materials is shown in Figure 3a. The pure BC membrane showed almost no catalytic ability for the degradation of TC. After loading a portion of 1T-2H MoS_2_, the BC-M membrane exhibited an improvement in adsorption performance, reaching about 12.8%. After 60 min of light exposure, the degradation of TC achieved 44.0%. This can be attributed to the reaction between Mo^4+^ and H_2_O_2_, which generates a certain amount of hydroxyl radicals (∙OH) [5,43]. Comparing the TC degradation curves of BC-M and BC-MP, it is evident that the loading of PPy significantly improved the catalytic degradation efficiency (approximately 77.5%). This can be attributed to the loading of PPy, which alters the band structure of 1T-2H MoS_2_, promoting the separation of photo-generated electrons and holes. This, in turn, enhances the conversion between Mo^6+^ and Mo^4+^, facilitating the reaction between Mo^4+^ and H_2_O_2_, which ultimately improves the TC degradation efficiency. By comparing the TC degradation curves of BC-MP and BC-MT, it can be observed that the introduction of the polyphenol and iron coupling structure significantly improves the catalytic rate of the composite membrane material. Furthermore, the BC-MTP membrane achieved 86.1% degradation of TC in just 30 min, with a maximum degradation efficiency of 88.2%. Figure 3b illustrates the effect of different TP and PPy loading amounts on the catalytic performance of the composite membrane materials. When the loading of the copolymer is too low, the degradation rate of the BC-MTP membrane is reduced due to insufficient iron source, and the final degradation efficiency is also weakened. On the other hand, when the copolymer loading is too high, an excess of ∙OH is generated, which reacts with H_2_O_2_ and ultimately lowers the TC degradation efficiency.

Figure 3c explores the importance of H_2_O_2_ in the degradation of TC by the BC-MTP membrane by varying the amount of H_2_O_2_ added. When no H_2_O_2_ is added, the degradation efficiency of TC by the BC-MTP membrane is only 24.0%. As the amount of H_2_O_2_ increases to 10 µL, the degradation efficiency of TC significantly improves, reaching 88.2%. At an H_2_O_2_ dosage of 20 µL, the maximum degradation efficiency does not increase further, whereas the degradation rate of the BC-MTP membrane is improved. However, when the H_2_O_2_ dosage is further increased to 30 µL, the degradation efficiency decreases. This decrease can be attributed to the excessive H_2_O_2_, which promotes self-reactions and consequently reduces its effective utilization in the degradation process. In addition, compared to the optimal H_2_O_2_ addition amount (80 µL) in previous studies for MTP in TC degradation [9], the optimal H_2_O_2_ addition amount for BC-MTP (20 µL) is significantly lower. This could be attributed to the catalyst being immobilized on the BC membrane, which reduces the leaching of Fe, thus improving the utilization of H_2_O_2_ and reducing its consumption. Furthermore, the BC-MTP membrane achieves the maximum degradation efficiency (88.2%) of TC in 60 min, while the MTP powder achieves its maximum degradation efficiency (86.3%) in 30 min. This result indicates that the degradation rate of the BC-MTP membrane is significantly slower than that of MTP powder, which can be attributed to the lower Fe content in the solution, slowing down the overall degradation process. However, a higher TC degradation efficiency can still be achieved with lower H_2_O_2_ consumption. This highlights the importance of constructing catalytic membrane materials to improve H_2_O_2_ utilization.

**Figure 4:**
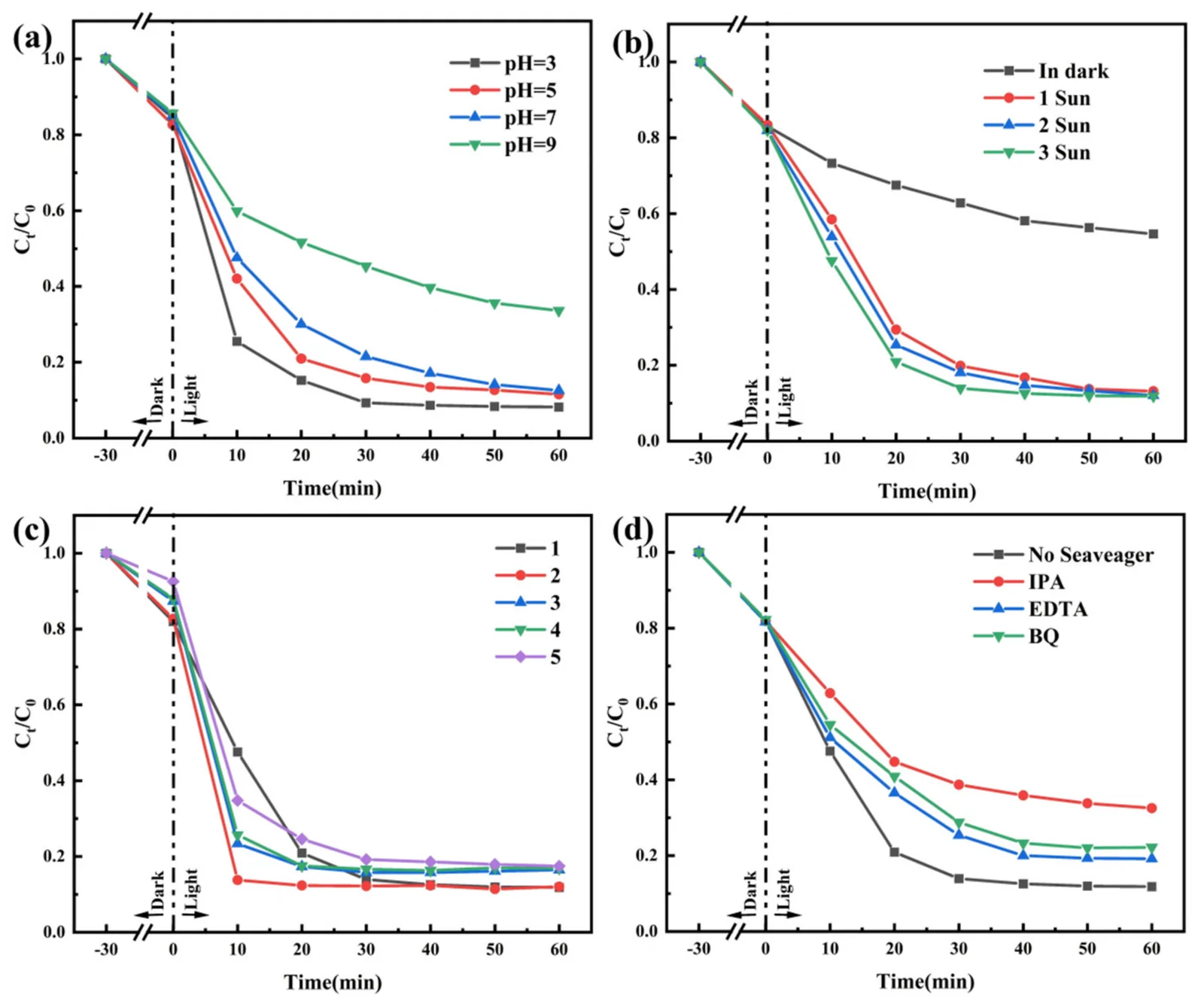
(**a**) Photo-Fenton degradation of TC by BC-MTP under different solution pH conditions; (**b**) Photo-Fenton degradation of TC by BC-MTP under different light intensities; (**c**) Photo-Fenton cycle performance of BC-MTP for TC degradation; (**d**) Radical scavenger detection.

Figure 4a illustrates the effect of solution pH on the degradation of TC by the BC-MTP membrane. As the pH increased, the degradation efficiency gradually decreased. The highest TC removal efficiency, approximately 91.8%, was obtained at pH 3, whereas the efficiency decreased to 66.4% at pH 9, indicating that the photo-Fenton reaction is more favorable under acidic conditions. It should be emphasized that these degradation curves reflect short-term catalytic performance rather than the chemical stability of the membrane. The retention of catalytic activity at pH 7 and pH 9 indicates that BC-MTP remains functional during the tested reaction period, but it does not demonstrate that the chemical composition and interfacial structure remain unchanged above pH 7. The available Supplementary (Figure S2) characterization after short-term treatment at pH 12 provides only limited information on changes in catalyst-related elemental composition and is insufficient to establish the long-term alkaline stability of the complete BC-MTP membrane. Under strongly alkaline conditions, surface oxidation, dissolution or loss of Mo/S-containing species, changes in Fe coordination, and alteration of the polymer/cellulose interface may occur. Therefore, BC-MTP is considered operationally functional under the tested neutral and mildly alkaline conditions, whereas its long-term chemical integrity under strongly alkaline conditions requires further verification by before/after structural characterization, mass-retention measurements, and quantitative analysis of dissolved Fe, Mo, and S.

In addition, the effect of light intensity on the catalytic performance of the BC-MTP membrane was investigated. As shown in Figure 4b, the membrane still exhibited a certain TC removal ability under dark conditions (45.4%). Upon irradiation, the TC degradation performance was substantially improved. However, the final degradation efficiency under one-sun irradiation (86.8%) was close to that obtained under the higher tested irradiation intensities (88.2%), indicating that increasing the light intensity within the investigated range produced only a limited improvement in the final removal efficiency.

The cycling experiment in Figure 4c evaluates retained catalytic function. After five cycles, the TC degradation efficiency decreased by 5.7%, while adsorption gradually declined, potentially because residual TC or intermediates occupied the membrane pores. Catalytic cycling alone does not prove coating-adhesion stability. Therefore, the morphology was additionally compared before and after five repeated water-washing cycles (Figure S3). The BC fiber network and MoS_2_-rich flower-like domains remained visible, with no obvious large-scale peeling or structural collapse. This observation supports short-term adhesion under the tested washing protocol, although it does not establish zero nanoscale component loss. Physical confinement by the interconnected BC network and the in situ TP/PPy-containing layer provide a plausible basis for immobilization. Radical-scavenging tests showed that IPA, EDTA, and BQ reduced TC degradation, supporting participation of •OH, h^+^, and •O_2_^−^, with •OH exerting the strongest effect.

**Figure 5:**
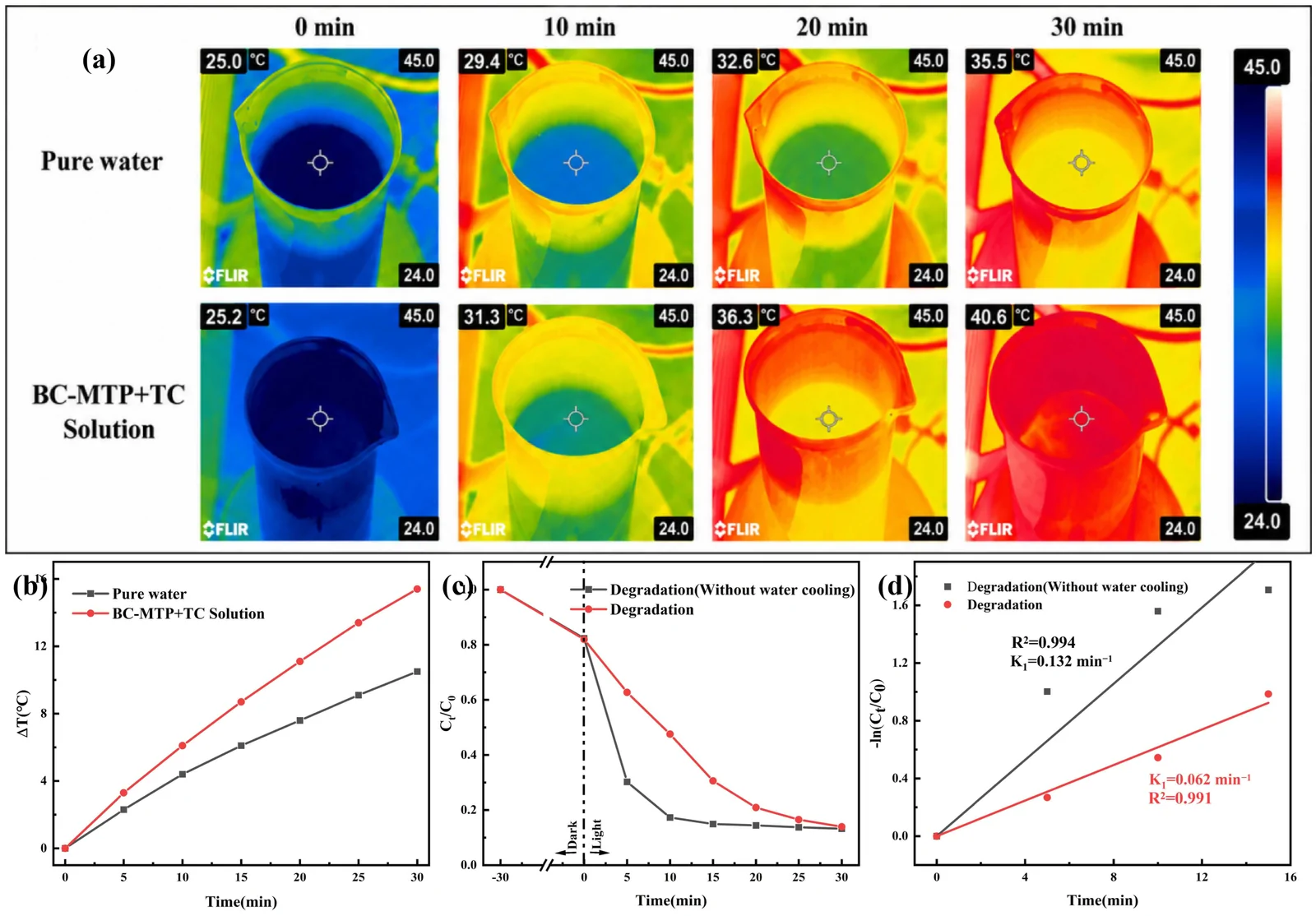
(**a**) Temperature variation of pure water and the BC-MTP-containing TC solution under irradiation; (**b**) corresponding temperature increase; (**c**) TC degradation under non-circulating photothermal-assisted and water-circulating approximately temperature-controlled conditions; and (**d**) corresponding kinetic comparison. Both reaction conditions were tested under the same incident light spectrum.

The non-thermostatized experiment was retained to evaluate BC-MTP as an integrated photothermal/photo-Fenton material under solar-like operation, where light-induced heating is part of the intended working condition. Under the same incident spectrum, membrane loading, TC concentration, H_2_O_2_ dosage, irradiation geometry, and reaction time, an external water-circulation experiment was used as an approximately temperature-controlled reference. As shown in Figure 5a,b, the non-circulating solution reached 40.6 °C after 30 min, and the TC-containing BC-MTP system showed a larger temperature increase than pure water. TC removal was faster without circulation, with apparent pseudo-first-order constants of 0.132 and 0.062 min^−1^ for the non-circulating and circulating conditions, respectively; the corresponding R^2^ values were 0.994 and 0.991, as shown in Figure 5c,d. This comparison indicates an apparent enhancement associated with bulk temperature rise under the tested conditions. It does not spectrally separate UV from visible-light photocatalysis because both experiments used the same incident spectrum. Temperature may influence H_2_O_2_ activation, Fe/Mo redox cycling, radical generation, mass transfer, adsorption/desorption, and diffusion.

**Figure 6:**
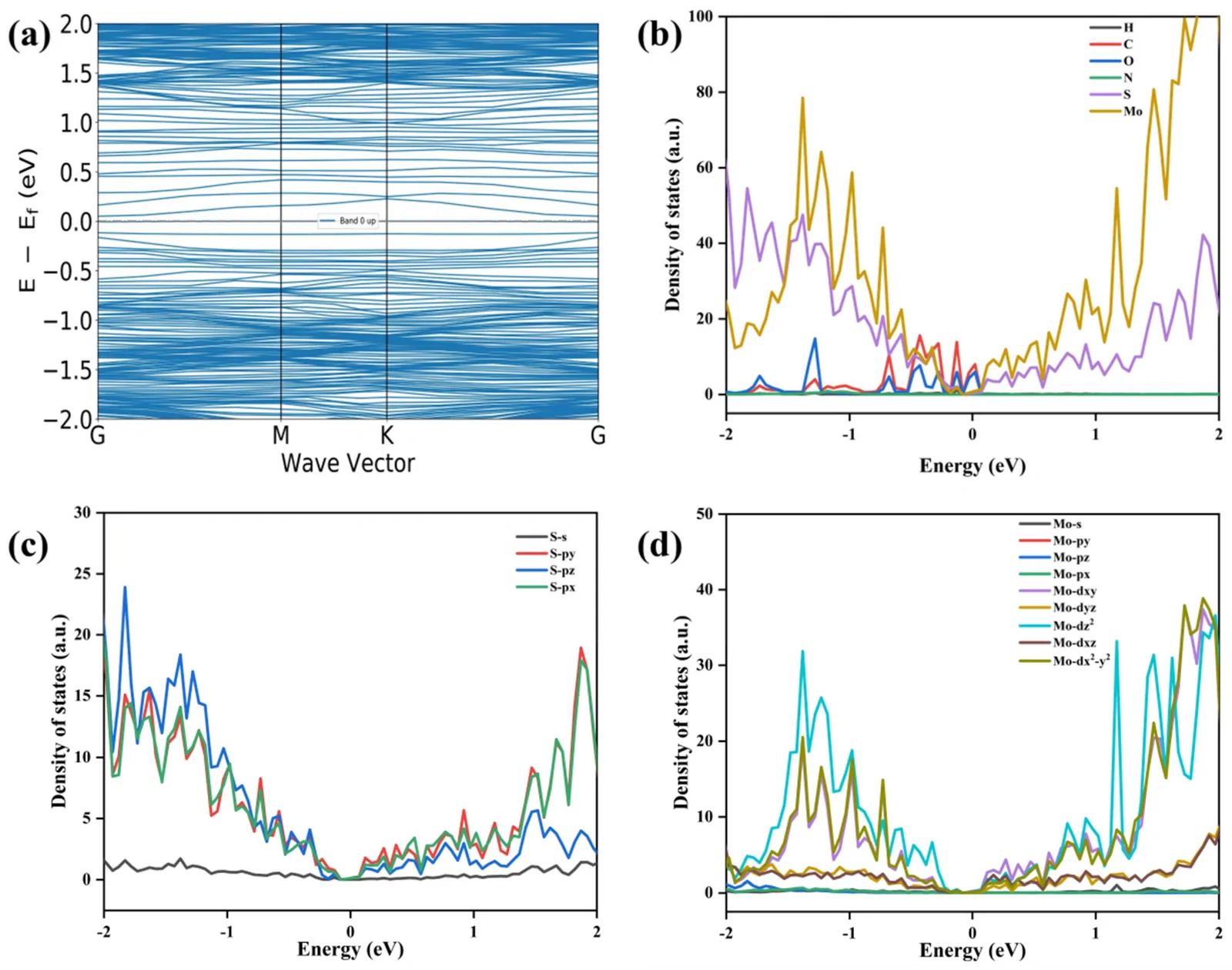
The band structure (**a**) and density of states (DOS) (**b**–**d**) of 1T-2H MoS_2_@TP/PPy.

The band structure and density of states (DOS) of the selected 1T-2H MoS_2_@TP/PPy model were calculated using the DS-PAW first-principles plane-wave software (DS-PAW 2023A) [44]. The calculated composite contains electronic states close to the Fermi level (Figure 6a), and the projected DOS indicates major contributions from Mo d and S p orbitals together with organic-component-derived states (Figure 6b). As shown in Figure 6c,d, the similarity of the Mo d and S p DOS profiles within approximately −1.5 to 1.5 eV is consistent with strong Mo–S electronic interaction [45,46,47]. However, equivalent calculations for pristine 1T-2H MoS_2_ and structurally defined TP/PPy reference models were not available. Consequently, the present result is used only as a qualitative description of orbital contributions within the final composite and is not interpreted as direct proof of heterostructure-induced bandgap narrowing or accelerated electron–hole separation.

### 2.3. Photothermal Water Evaporation Performance Study

**Figure 7:**
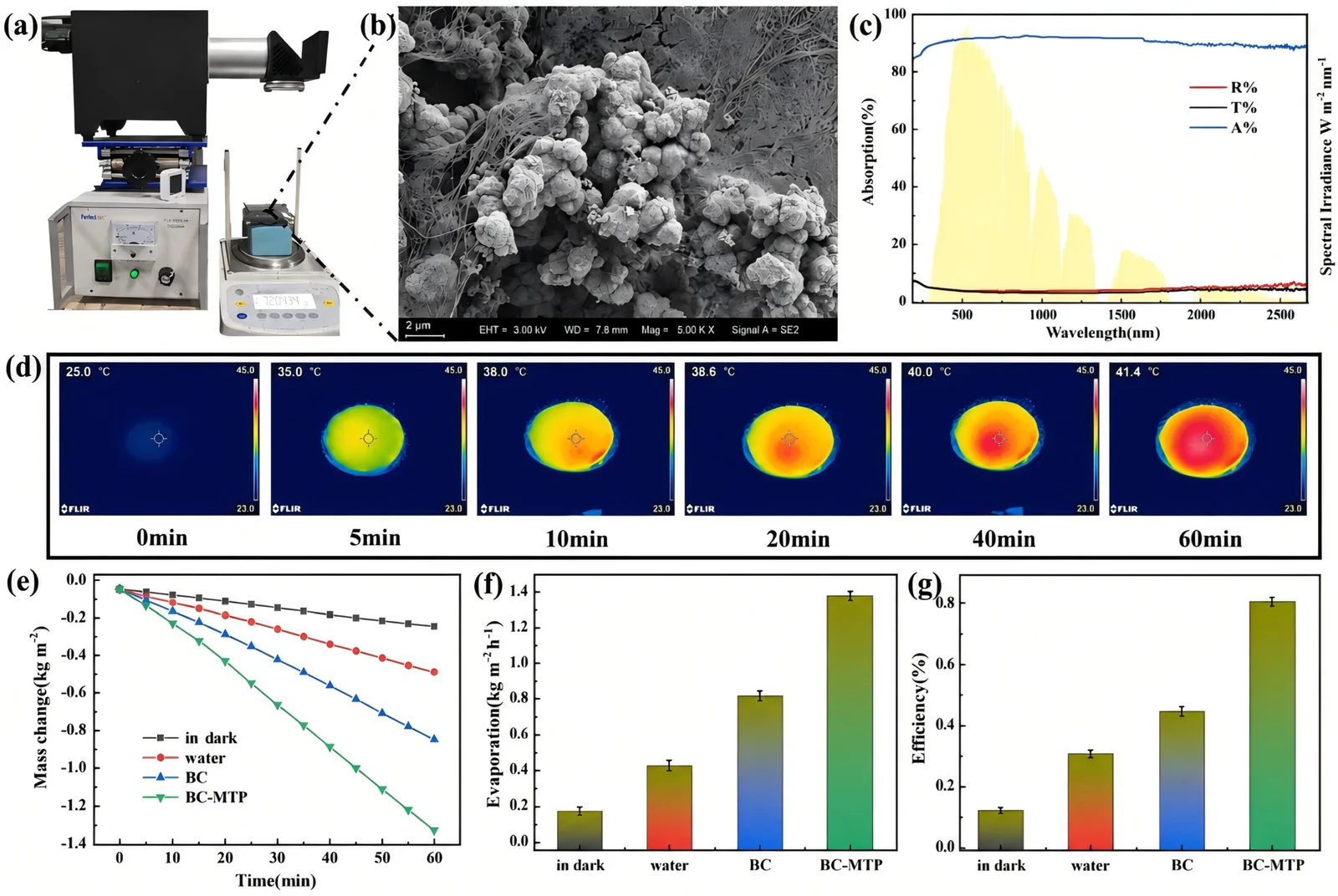
(**a**) Indoor simulated solar interface evaporation setup. (**b**) SEM image of the BC-MTP membrane. (**c**) Full-spectrum absorption curve of the BC-MTP membrane. (**d**) Surface temperature of the BC-MTP membrane. (**e**) Mass change of dark environment, water, BC, and BC-MTP membranes. (**f**) Evaporation rate and (**g**) Evaporation efficiency of the BC and BC-MTP membranes.

The laboratory interfacial-evaporation setup is shown in Figure 7a. The SEM image in Figure 7b uses the verified length unit μm and shows MoS_2_-rich clusters together with a locally thicker TP/PPy-containing layer, which may partially obstruct water-transport pathways. Figure 7c shows that BC-MTP has approximately 91% average absorptance over the measured spectral range (the yellow region represents the solar spectrum). Component-resolved control spectra are provided in Figure S4: pure BC exhibits substantially higher reflectance and lower absorption, whereas BC-M, BC-MP, and BC-MT show enhanced broadband harvesting after introduction of MoS_2_ and the conjugated organic components. BC-MTP displays the strongest overall absorption among the tested membrane samples, supporting the combined contribution of MoS_2_ and TP/PPy to light harvesting. Under one sun, the BC-MTP surface reached 41.4 °C after 60 min, corresponding to a 16.4 °C increase.

The interface water evaporation performance of the membrane was evaluated by calculating the evaporation efficiency (*η*) using the following formula [48,49]:

(2) $$\eta =\frac{∆m\times \left(He+Q\right)}{∆t\times Sum\times A}$$

(3) $$Q\;=c\;\times \;(T-T_{1})$$

where *η* is the evaporation efficiency, *He* is the evaporation enthalpy of water from liquid to gas (≈2400 kJ·kg^−1^), *Q* is the sensible heat of unit mass of water, Δ*m* is the mass change, Δ*t* is the time change, *Sum* is the solar light intensity (1 kW·m^−2^), *A* is the membrane area, *c* is the specific heat capacity of water (≈4.2 J·g^−1^ K^−1^), *T*_1_ is the initial surface temperature of the membrane material, *T* is the final surface temperature of the membrane material.

As shown in Figure 7f, the evaporation rate of the BC membrane is 0.81 kg·m^−2^ h^−1^, which is higher than the evaporation rate of pure water (0.43 kg·m^−2^ h^−1^). The evaporation rate of the BC-MTP membrane is 1.34 kg·m^−2^ h^−1^, which is significantly higher than both pure water and BC membranes, approximately 3.1 times higher than the evaporation rate of pure water. Figure 7g shows the corresponding evaporation efficiency. The evaporation efficiency of the BC membrane is 43.7%, while the evaporation efficiency of BC-MTP is 80.1%. The evaporation efficiency of the BC-MTP membrane shows a significant improvement.

## 3. Materials and Methods

### 3.1. Chemicals and Materials

Ferric(III) chloride hexahydrate (FeCl_3_·6H_2_O, AR, 99%), hydrogen peroxide solution (H_2_O_2_, AR, 30 wt% in H_2_O), pyrrole (Py, C_4_H_5_N, 99%), ammonia (H_5_NO, AR, 25–28%) and Tetracycline (TC, C_22_H_24_N_2_O_8_, AR) were purchased from Shanghai Aladdin Biochemical Technology Co., Shanghai, China. Tea polyphenols (TP, 99%) were purchased from Shanghai McLean Biochemical Technology Co., Shanghai, China. Bacterial cellulose (BC) was purchased from Hainan Yide Food Co., Haikou, China.

### 3.2. Preparation of 1T-2H MoS_2_

To obtain black 1T-2H MoS_2_ powder, we synthesized 1T-2H MoS_2_ via a one-step hydrothermal method [9]. To synthesize black 1T-2H MoS_2_ powder, we initiated the process by dispersing 2 mmol of sodium molybdate dihydrate (Na_2_MoO_4_-2H_2_O), along with 6 mmol of thiourea (CS(NH_2_)_2_), and 16 mL of propionic acid (C_3_H_6_O_2_) in 70 mL of deionized water. This mixture underwent sonication to attain a homogenously dispersed solution. Subsequently, the solution was transferred into a PTFE-lined autoclave and sealed for the reaction. The reaction was carried out in a water bath at a temperature of 180 °C for a duration of 4 h. Following the completion of the reaction, the autoclave was allowed to gradually cool down to room temperature. The resulting mixture was then subjected to filtration, with several rinses using deionized water and anhydrous ethanol. Lastly, the product obtained from the reaction was dried using a vacuum drying oven, maintaining a temperature of 60 °C over a period of 12 h. This meticulous process resulted in the production of black 1T-2H MoS_2_ powder, achieved through the subsequent grinding of the dried product.

### 3.3. Preparation of BC/1T-2H MoS_2_@TP/PPy Composite Material

First, 2 g of BC was sonicated in 100 mL of deionized water for 4 h using an ultrasonic cell disruptor to obtain a dispersed BC solution. Subsequently, 100 mg of 1T-2H MoS_2_ was dispersed into the BC solution through magnetic stirring and ultrasonication, followed by continuous stirring for 30 min. By ultrasonication, 0.02 g of TP and 0.13 g of FeCl_3_·6H_2_O were dispersed in 4 mL and 10 mL of deionized water, respectively. Subsequently, the ultrasonicated TP solution was added dropwise, followed by the addition of 0.1 mL of Py monomer, resulting in a uniformly dispersed solution, referred to as Solution A. Subsequently, the FeCl_3_·6H_2_O solution was gradually added dropwise to Solution A under continuous stirring. Finally, the pH of the solution was adjusted to 9 using 28% ammonia water. The reaction was allowed to proceed for 1 h in a low-temperature polymerization setup at 5 °C. The resulting mixture was then filtered and washed multiple times with deionized water and absolute ethanol. Afterward, the product was dried at room temperature for 12 h to obtain the BC-MTP composite membrane.

In addition, BC-1T-2H MoS_2_ (BC-M) was prepared using the same procedure without adding any copolymers. Similarly, BC-1T-2H MoS_2_@PPy (BC-MP) or BC-1T-2H MoS_2_@TP (BC-MT) was synthesized by excluding the TP solution or Py monomer, respectively. To determine the optimal copolymer ratio, various proportions of TP, Py monomer, and FeCl_3_·6H_2_O were introduced to synthesize the MTP composite catalysts, while keeping the mass of BC and 1T-2H MoS_2_ constant. Under a fixed mass ratio of TP, Py monomer, and FeCl_3_·6H_2_O, composite catalysts named BC-MTP-0.5, BC-MTP-1, and BC-MTP-2 were prepared with 1T-2H MoS_2_ to Py mass ratios of 1:0.5, 1:1, and 1:2, respectively.

### 3.4. Material Characterization

Scanning electron microscopy (SEM, SM-6700F, JEOL Ltd., Tokyo, Japan) was used to characterize the micromorphological structure of the membrane materials. X-ray diffractometer (XRD X’Pert PRO, PANalytical B.V., Almelo, The Netherlands) was operated in the 5–90° 2θ range at 40 mA and 40 kV to obtain the crystal structure of the materials. An X-ray photoelectron spectrometer (XPS Thermo ESCALAB 250XI, Thermo Fisher Scientific, Waltham, MA, USA) was employed to characterize the surface elements and their chemical state of the membrane materials. Raman spectra of the composite membrane materials were also obtained using a Raman spectrometer (M-30-TPM, Bunko-Keiki Co., Ltd., Tokyo, Japan). Xenon light source (PLS-SXE300, Beijing Perfectlight Technology Co., Ltd., Beijing, China) is used to perform simulated light sources for simulated sunlight evaporation experiments. A thermal imaging camera (FLIR E60, FLIR Systems, Inc., Wilsonville, OR, USA) was used to record the temperature data for the photothermal tests.

### 3.5. Photothermal-Enhanced Fenton Performance Test

The degradation experiment was conducted using a 300 W xenon lamp (PLS-SXE300, Beijing Perfectlight Technology Co., Ltd., Beijing, China) for irradiation, with the catalyst dispersed in 200 mL of tetracycline (TC, 20 mg/L) aqueous solution. Prior to light exposure, the solution was stirred in the dark for 30 min to establish an adsorption–desorption equilibrium. Different amounts of catalyst were added and evaluated to determine the optimal loading. Similarly, the copolymer and H_2_O_2_ concentrations were varied to determine the most suitable loading and optimal H_2_O_2_ dosage. The effect of light intensity on the photocatalytic Fenton degradation process was studied by adjusting the intensity of the xenon lamp. The impact of pH on the degradation process was investigated by altering the pH of the solution.

During the degradation process, the temperature was recorded every minute, and infrared thermal images were captured. Additionally, 4 mL of the solution was collected every 10 min, centrifuged at 8000 rpm, and the concentration of TC in the solution was determined using a UV–Vis spectrophotometer (UV-2600, Shimadzu Corporation, Kyoto, Japan) by measuring absorbance at 664 nm and 357 nm. It is important to note that the entire experiment was conducted under visible light conditions, with a 420 nm cutoff fluorescence spectrophotometer used to ensure that the photocatalytic Fenton degradation occurred within the visible light parameter range.

### 3.6. Room Temperature Interfacial Water Evaporation Test

The melamine sponge was trimmed into a disc with a diameter of 3 cm and a thickness of approximately 2 cm. Then, the sonicated bacterial cellulose was flocculated and attached to the melamine sponge. After allowing the sample to dry naturally at room temperature, it was placed into a beaker containing deionized water to ensure adequate humidity. This setup was used as the water transport layer and thermal insulation layer. The wetted sample (3 cm in diameter) was placed flat on the BC-coated melamine sponge. The remaining space was filled with foamed foam, and the setup was covered with aluminum foil to prevent the effects of water self-evaporation. The sample was then placed under a solar simulator for irradiation to evaluate its evaporation characteristics.

The mass change of water was recorded every 5 min using an electronic balance with an accuracy of 0.001 g to calculate the evaporation rate. The surface temperature change of the sample was monitored using an infrared camera. The intensity of the simulated sunlight (1000 W·m^−2^) was adjusted using a power meter. The power meter was used to calibrate the simulated sunlight intensity to 1000 W·m^−2^. The ambient temperature and humidity were approximately 25 °C and 60%, respectively.

## 4. Conclusions

In this study, BC was used as a substrate, and 1T-2H MoS_2_ powder and TP/PPy copolymer were successfully loaded onto the BC substrate via blending and copolymerization methods. The overall molecular structure of BC and the hybrid phase of MoS_2_ were retained, addressing the issue of catalyst recovery. The photocatalytic-Fenton degradation results showed that the BC-MTP membrane exhibited excellent degradation performance for TC. The BC-MTP membrane consumed significantly less H_2_O_2_ (20 µL) compared to MTP (80 µL). This can be attributed to the BC substrate, which effectively fixed the iron source, reducing the leaching of iron and thereby improving the utilization of H_2_O_2_. Moreover, the BC-MTP membrane demonstrated excellent cyclic stability, with only a 5.7% decrease in overall degradation efficiency after five cycles, indicating its durability and reliability. In indoor water evaporation simulation experiments, the MTP material exhibited a certain degree of photothermal performance. Compared to the evaporation efficiency of the pure BC membrane (43.7%), the water evaporation performance of the BC-MTP composite membrane was significantly improved, reaching 80.1%. Therefore, this method provides a promising and sustainable solution for catalyst recovery.

## Data Availability

The original contributions presented in this study are included in the article/Supplementary Material. Further inquiries can be directed to the corresponding authors.

## Supplementary Materials

The following supporting information can be downloaded at: https://www.mdpi.com/article/10.3390/molecules31162878/s1, SEM images of BC-MTP (Figure S1). SEM images and energy-dispersive spectroscopy (EDS) mappings of MTP after suction-desorption in solution at pH = 12 for 30 min (Figure S2). (a) SEM image of the BC-MTP membrane; (b) SEM image of the BC-MTP membrane after 5 washing cycles. (Figure S3). (a) Full-spectrum absorbance and reflectance of BC; (b) Full-spectrum absorbance and reflectance of BC-M, BC-MT, BC-MP, and BC-MTP (Figure S4). Weight percentage and atomic percentage of MTP (Table S1). Weight percentage and atomic percentage of MTP after suction desorption in pH = 12 solution for 30 min (Table S2).
