# Supplementary material for: Fabrication of a BC/1T-2H MoS2@TP/PPy Membrane for Photo-Fenton Reactions and Solar-Driven Water Evaporation
Source: Molecules. 2026 Aug 18;31(16):2878. doi: 10.3390/molecules31162878 (PMC13515191; doi:10.3390/molecules31162878)
Supplement: Supplementary file 1 [file molecules-31-02878-s001.zip › molecules-4408075-supplementary.pdf]

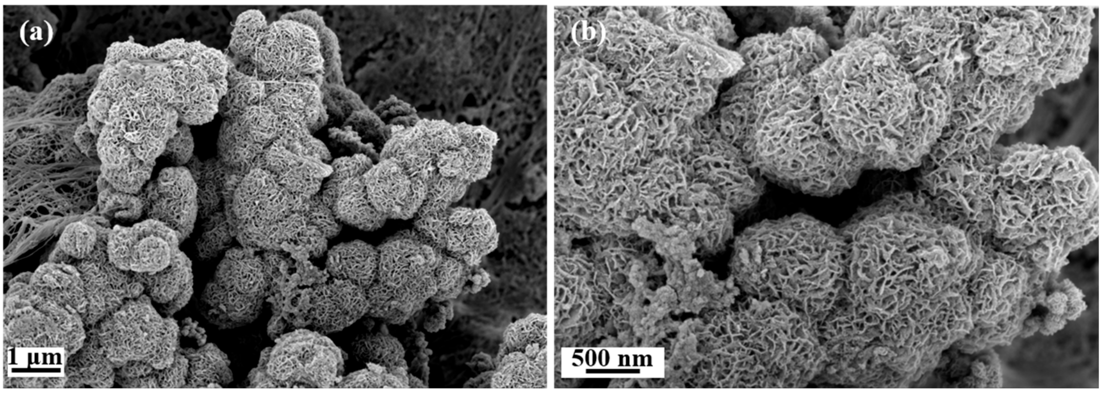

Figure S1. SEM images of of BC-MTP.

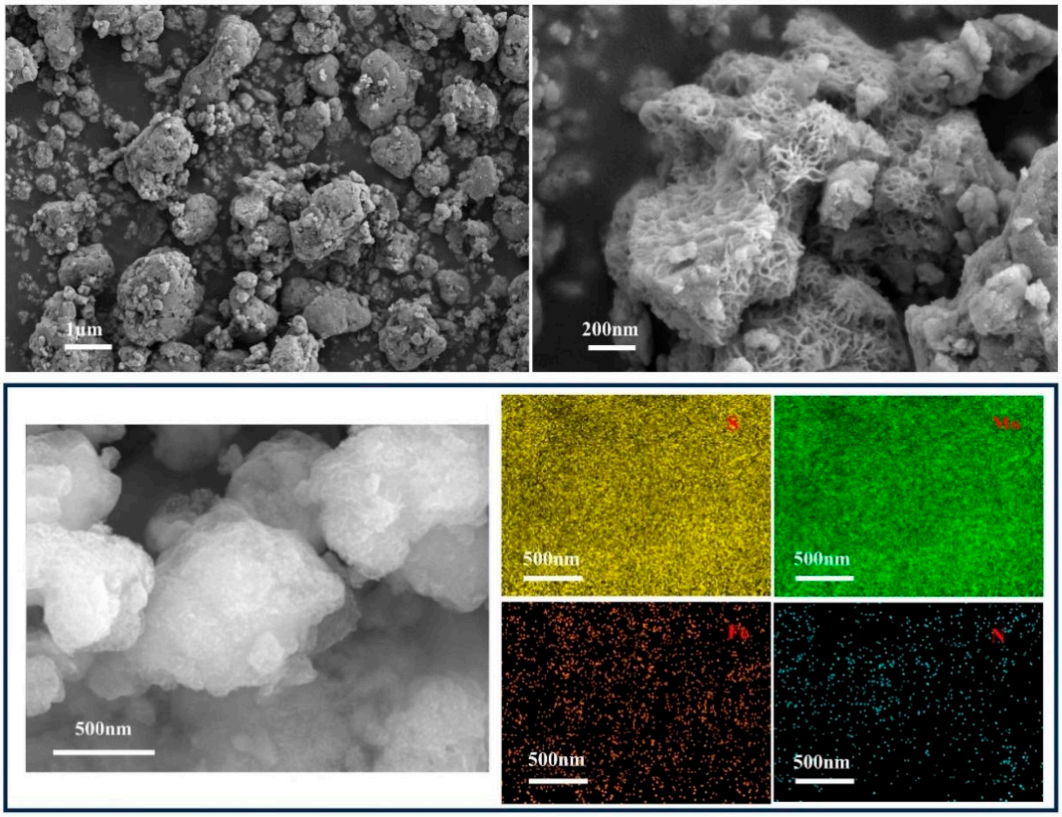

Figure S2. SEM images and energy dispersive spectroscopy (EDS) mappings of MTP after suction-desorption in solution at pH=12 for 30 min.

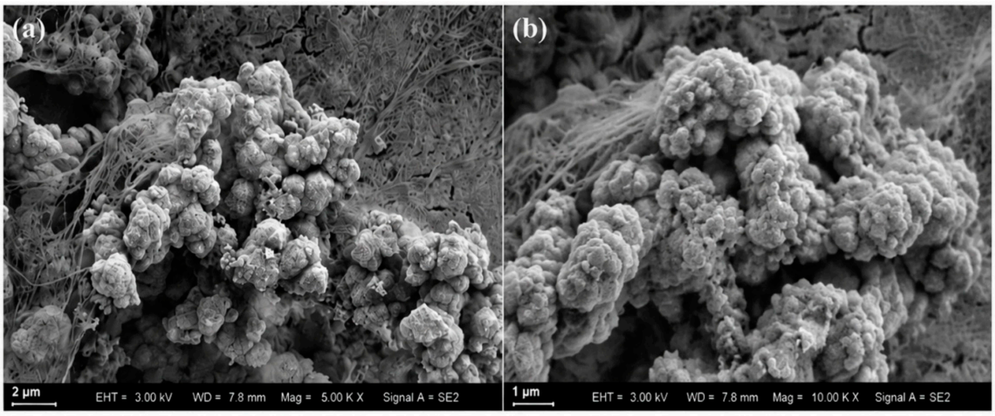

Figure S3 (a) SEM image of the BC-MTP membrane; (b) SEM image of the BC-MTP membrane after 5 washing cycles.

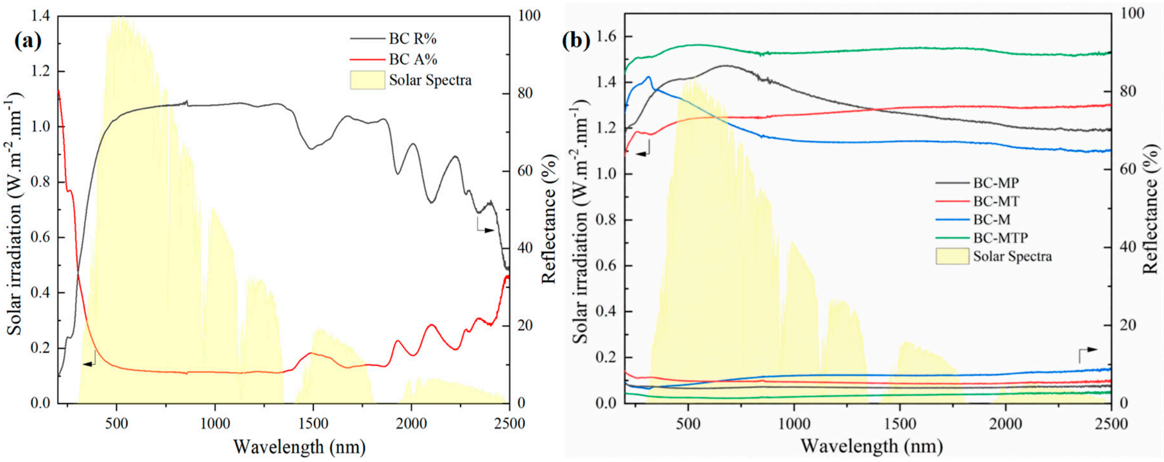

Figure S4 (a) Full-spectrum absorbance and reflectance of BC; (b) Full-spectrum absorbance and reflectance of BC-M, BC-MT, BC-MP, and BC-MTP.

Table S1 Weight percentage and atomic percentage of MTP

Element	Wt%	Percentage of atoms%
N	0.56	1.25
Mo	46.52	15.09
S	30.05	29.18
Fe	0.70	0.39

Table S2 Weight percentage and atomic percentage of MTP after suction desorption in pH=12 solution for 30 min.

Element	Wt%	Percentage of atoms%

N	1.28	2.45
Mo	41.34	11.52
S	26.95	22.51
Fe	1.38	0.66
